# Workplace violence: A complex challenge demanding a systemic response

**DOI:** 10.5271/sjweh.4249

**Published:** 2025-09-01

**Authors:** Sofie Jaspers, Iben Karlsen, Birgit Aust

**Affiliations:** 1 The National Research Centre for the Working Environment, Lersø Parkallé 105, 2100 Copenhagen, Denmark.

Workplace violence is associated with negative consequences for workers, organizations, and society. Experiencing violence at work has consistently been associated with an increased risk of symptoms of depression and anxiety, diagnosis of post-traumatic stress disorder (PTSD), and suicide (1–3). Further, workplace violence has been found to increase the risk of physical health outcomes such as type 2 diabetes and cardiovascular diseases (4, 5). Workplace violence can also lead to higher rates of turnover and sick leave (1, 6), translating into a societal economic burden (7).

Longitudinal studies indicate that repeated exposure to workplace violence, compared with single episodes of violence, increases the risk of PTSD and sick leave further (8–10). Moreover, a recent study shows synergistic interaction effects for simultaneous exposure to workplace violence and other psychosocial working conditions, such as high emotional demands and high quantitative demands—conditions that are typically present in the same sectors (such as healthcare and education) where violence is frequently reported (11).

## Prevalence of workplace violence – the problem persists

In a 2022 global survey, the International Labor Organization (ILO) found that more than one-fifth of the workforce experienced violence and harassment at work during their working life (12). While this gives an idea of the magnitude of the problem, it covers large differences between countries and sectors, as well as among types of violence and harassment. Effective prevention requires a more precise understanding of all these aspects, but in many countries, comprehensive data to identify the most exposed groups are not available. For Europe, however, it is possible to describe the main characteristics of workplace violence – which may be quite different from other parts of the world (12). Estimates from Europe indicate that 2–5% of the general workforce is affected by work-related violence, with substantially higher rates—ranging from 5–30%—reported in high-risk sectors (13–16). Despite these overall trends, comparing the prevalence of workplace violence remains challenging due to variations in definitions and measurements; in addition, widespread underreporting makes it difficult to obtain accurate figures (17).

In this issue of the *Scandinavian Journal of Work, Environment and Health*, a longitudinal study by Gash & Blom (15) addresses some of these issues. Using nationally representative data from the UK, the authors show that workplace violence is prevalent in a wide variety of sectors, but with elevated numbers in public administration (eg, benefits administration), followed by health- and residential care and social work.

Because the study by Gash & Blom uses the same set of questions to assess violence across sectors, patterns can be identified, making it possible to compare them with results from similar studies. Two relevant cohorts to compare their results with are the Work Environment and Health in Denmark study (WEHD) (18) and the European Survey of Enterprises on New and Emerging Risks (ESENER) conducted by Eurofound (13, 14). The WEHD study comprises identical measures of violence at work in four surveys of national samples of the Danish working population conducted between 2012 and 2018. The three sectors persistently reporting the highest levels of violence are healthcare, social work, and education (16, 19). The same trend is seen in the ESENER survey, where health and social work, education, and public administration were the top three most exposed sectors in both 2015 and 2021 (13, 14).

Together, data reveal a persistent and concerning pattern for European countries, showing that workplace violence remains particularly high in specific sectors. As evidence of both the prevalence and consequences of the problem continues to grow, the need for decisive action becomes increasingly urgent.

## From problem to strategy: making the case for systemic solutions

Workplace violence can be categorized into three types, each guiding tailored prevention efforts (20). Type I involves perpetrators with no legitimate connection to the workplace—typically criminal acts such as robberies. Type II arises from interactions with clients, patients, or service users, where violence occurs without criminal intent. Type III refers to violence from current or former colleagues, supervisors, or acquaintances within the organization (20).

Cross-national patterns in the sectors most affected by workplace violence (healthcare, social work, and education) suggest that violent incidents are associated with a high degree of contact with clients, patients, or service users, characteristic of Type II violence. Type II violence typically arises in situations where the “perpetrator” experiences emotional stress caused by pain or frustration due to, eg, rejection of requests perceived as legitimate (21, 22). While the term violence is meaningful from an occupational safety and health (OSH) perspective, it connotes an overly intentional act from the perpetrator that is often not the case. For example, a care worker in a residential facility may frequently encounter violent episodes involving elderly residents with severe behavioral symptoms of dementia. Preventive measures in these sectors must therefore include a deeper understanding of how to improve the well-being of the affected individual—both to protect the care worker and to enhance the quality of life for the resident (23).

Consequently, it is important to recognize that the prevention of Type II violence should be approached from a dual perspective—one that reflects a shared interest between the worker and the potential “perpetrator” in preventing conflict situations that normally precede the violent incident. This applies whether the source of the violent incident is a patient, a child in kindergarten, or a citizen who needs support from social services (23–25). As such, the prevention of violence is closely linked to professional practices within a given sector that aim to strengthen relational work with patients, clients, or service users.

As mentioned above, the patterns and characteristics of workplace violence may be different in other parts of the world, where Type I and Type III violence are more prevalent, and investigating these patterns would be important for tailored prevention.

## Policy measures and workplace interventions

While national and transnational legislation in the area has evolved with violence as a highly regulated psychosocial hazard (19, 26), that in itself does not seem to be enough to solve the problem (27, 28).

Recent research indicates that both external pressure from legislation and internal pressure on actors within organizations through OSH systems and procedures are needed to change practices to prevent psychosocial risks (29). Therefore, local intervention still carries significant importance.

Despite a rise in the number of intervention studies on workplace violence, only a few have succeeded in reducing workplace violence. Numerous reviews show that the majority of studies focus on de-escalation training, which seems to improve workers’ knowledge and self-efficacy, but has only limited or no impact on reducing violent incidents at work (17, 30–32).

The lack of effective preventive interventions might be explained by the multicausal nature of violent episodes, where risk factors on different levels interact to create the specific risk (33–35). Some studies have examined comprehensive multi-stranded interventions addressing this complexity, and the limited evidence available suggests these interventions can be effective (36–40).

However, these complex interventions can be especially challenging to conduct in workplaces that need them most. The high prevalence of workplace violence can considerably strain organizational resources, as such incidents are closely linked to increased rates of sickness absence and staff turnover (1, 6, 21, 41–43). The most vulnerable workplaces are therefore often caught in a downward spiral of resource depletion.

## A complex challenge demanding a systemic response

**Figure 1:**
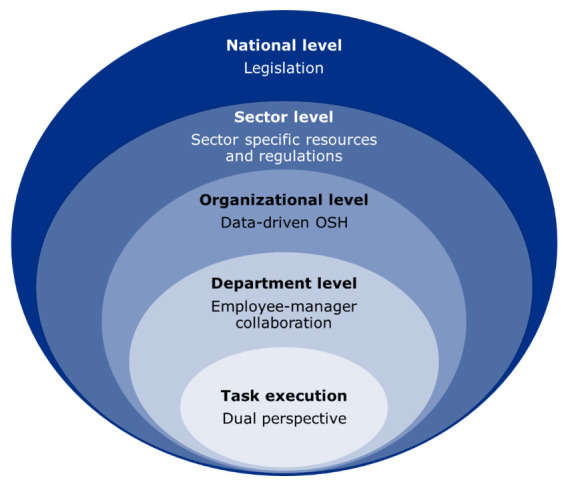
A systemic approach to workplace violence prevention

This situation calls for addressing broader systemic factors that support workplaces and sectors with the highest risks and the fewest resources—factors that currently hinder their ability to comply with regulatory requirements or successfully conduct workplace interventions. Inspired by systems thinking (44), we propose a systemic approach to workplace violence prevention, as represented in figure 1. Such a systemic approach enables the identification of gaps across organizational preventive practices and research knowledge and supports the recognition of key leverage points within the system that influence multiple interrelated challenges (44). The approach aligns with system-oriented approaches such as AcciMap analysis (45), which emphasize the importance of identifying the broader organizational and systemic factors that contribute to incidents of workplace violence. Rather than focusing solely on the safety behavior of frontline staff, such approaches highlight how decisions and interactions across multiple system levels—from policy to management to task execution—shape the conditions in which violence occurs.

The application of a systemic approach to workplace violence prevention is informed by studies from Australia in two high-risk sectors: healthcare and social work (32, 35), and violence-prevention research from Denmark in the high-risk sectors of eldercare, psychiatry, prison and probation services, and the education sector (23, 38, 46). The figure illustrates how violent episodes arise in the inner circle at the task execution level. However, task execution is nested within a broader context, affecting the situational risk. On the task execution level, the figure highlights the importance of adopting a dual perspective on conflict reduction—addressing both the employees’ work environment and the well-being of individuals who may pose a risk of violent situations. In healthcare, social work, and educational settings, violent episodes can create emotional and cognitive dilemmas—especially when acts are unintentional or where incrimination, eg, of an adolescent, may hinder the pedagogical relationship. Therefore, a dual perspective is needed, integrating conflict prevention and client well-being into occupational safety and health efforts (23, 34, 37). At the department level, preventive work should focus on strong collaboration between employees and managers to foster a safe and supportive psychosocial work environment (38). Psychosocial factors such as trust are essential for encouraging reporting and facilitating crucial knowledge sharing (24). At the organizational level, the focus should be on systematic, data-driven OSH efforts and policies, as typically advocated in the safety climate literature (36, 38). The two outer circles emphasize the significance of sector-specific resources and regulations, as well as (trans-)national legislation, in providing a solid framework for violence prevention (35, 47).

The systemic approach to violence prevention provides a valuable framework for organizing and critically assessing existing knowledge in the field. Applying this perspective reveals a notable gap in the literature: few studies address sector-level dynamics, despite their potential as key leverage points for systemic change. Decisions related to well-known implementation barriers—such as staffing and resource allocation—can be influenced at this level. A recent study from the Australian social work sector demonstrated how systemic risk factors can be mapped effectively through active engagement of stakeholders across all levels, bridging silos of OSH, visitation processes, and quality improvement (47).

This type of sector-level intervention that engages stakeholders beyond the OSH domain holds promise as an impactful strategy, given that decision-making authority over systemic factors often resides at this level. Such interventions offer a strategic opportunity to support the workplaces most in need. Adopting a systems perspective on workplace violence, therefore, requires coordinated action from multiple actors such as sector-specific interest organizations, employer organizations, and politicians.
